# Transrectal Biplane Ultrasound Combined with Cotton Swab Marking of the External Urethral Orifice for Evaluating the Postoperative Position of Transobturator Mid-Urethral Sling: A Study on Standardized Ultrasound Measurement and Inter-Observer Reliability

**DOI:** 10.3390/diagnostics16172864

**Published:** 2026-09-06

**Authors:** Xiaokun Li, Niya Wei, Ruijie Sun, Xinting Liu, Ying Zou, Xiaoyan Wei, Yuan Li, Yue Wang

**Affiliations:** Department of Ultrasound, Peking University Shenzhen Hospital, Shenzhen 518036, China; lixiaokun@pkuszh.com.cn (X.L.); weiniya@pkuszh.com.cn (N.W.); sunruijie@pkuszh.com.cn (R.S.); liuxinting@pkuszh.com.cn (X.L.); yingzou@pkuszh.com.cn (Y.Z.); weixiaoyan@pkuszh.com.cn (X.W.); liyuan@pkuszh.com.cn (Y.L.)

**Keywords:** transrectal biplane ultrasound, cotton-swab marking, transobturator mid-urethral sling, stress urinary incontinence, pelvic floor ultrasound, interobserver agreement

## Abstract

**Objectives**: To assess the technical feasibility and interobserver reliability of transrectal biplane ultrasound with external cotton-swab marking of the urethral orifice after transobturator mid-urethral sling surgery, and secondarily to describe dynamic measurements and exploratory associations with chart-derived postoperative leakage. **Methods**: In this single-center retrospective cross-sectional imaging study, 112 consecutive women examined between January 2024 and February 2026 underwent a standardized protocol. The swab tip was placed lightly against, but not inserted into, the external urethral orifice. Sling width (W), distance from the sling upper edge to the internal urethral orifice (D-I), and distance from the sling lower edge to the external urethral orifice at rest (D-O) and during Valsalva (V-D-O) were measured. Forty randomly selected examinations were independently measured by two blinded readers. Agreement was evaluated using ICC(2,1), Bland–Altman bias and 95% limits of agreement (LoA), standard error of measurement (SEM), and minimal detectable change (MDC95). Missingness patterns and age-adjusted exploratory logistic models were examined. **Results**: W, D-I and D-O were measurable in all 112 women; V-D-O was available in 95 (84.8%). Postoperative leakage status was available in 101 women (62 without and 39 with leakage). V-D-O was shorter than D-O in paired examinations (median difference, −3.00 mm; 95% CI, −4.90 to −1.00; *p* < 0.001). ICCs were 0.845 for W, 0.942 for D-I, 0.936 for D-O and 0.970 for V-D-O. Bland–Altman biases (reader A minus reader B) were −0.18, −0.93, 0.70 and −0.23 mm, respectively; corresponding MDC95 values were 1.53, 2.96, 3.72 and 2.33 mm. Missing V-D-O was associated with a shorter postoperative interval after Holm correction. None of six age-adjusted ultrasound models was associated with chart-derived leakage after Holm correction. **Conclusions**: The protocol permitted highly available static measurements and good-to-excellent interobserver reliability. Because the study lacked an unmarked comparator, intraobserver repeats and a standardized clinical endpoint, it supports technical feasibility and measurement reliability, not superiority, diagnostic validity or prognostic utility.

## 1. Introduction

Stress urinary incontinence (SUI) is a common female pelvic floor disorder that can substantially impair quality of life. For women with bothersome symptoms who do not respond to, or are unsuitable for, conservative treatment, mid-urethral sling surgery remains an important option. Tension-free vaginal tape-obturator (TVT-O) is widely used because it is minimally invasive and follows a well-defined surgical route [1,2].

Postoperative outcomes after TVT-O are generally evaluated using symptoms, cough-stress testing, pad testing, urodynamic assessment or validated patient-reported measures [1]. Clinical assessment, however, cannot directly depict the sling’s spatial relationship with the urethra, bladder neck, anterior vaginal wall and adjacent structures. Transperineal/translabial ultrasound and transvaginal ultrasound are the established first-line sonographic routes for sling visualization and can depict the sling and neighboring soft tissues dynamically [3,4,5,6].

Previous studies have used mainly transperineal/translabial or transvaginal ultrasound to assess sling position, mobility and associations with continence outcomes or lower urinary tract symptoms [5,6,7,8,9,10,11]. Scanning routes, anatomical reference points, measurements and clinical endpoints nevertheless vary substantially [11]. A reproducible distal urethral reference point may facilitate measurement, but the external urethral orifice can be indistinct on conventional pelvic floor ultrasound. Few studies have evaluated an external physical marker as a landmark; importantly, such a method requires direct methodological validation before any claim of superiority can be made.

High-frequency transrectal biplane ultrasound can provide simultaneous sagittal and transverse views of the anterior vaginal wall, urethra and sling. Published reviews emphasize transperineal/translabial and transvaginal imaging for pelvic floor and sling assessment; transrectal biplane ultrasound has not been established as a first-line postoperative sling-imaging route and was therefore treated here as a complementary investigational examination [3,4,5,6,12]. We hypothesized that an externally positioned cotton swab could make the external urethral orifice consistently visible during this complementary examination and support repeatable offline distance measurements. The swab was never inserted into the urethral lumen.

We therefore evaluated a standardized protocol combining transrectal biplane ultrasound with external cotton-swab marking of the external urethral orifice. The primary objectives were to quantify technical feasibility, defined by measurement availability, and interobserver reliability. Secondary objectives were to describe rest-to-Valsalva changes and conduct explicitly exploratory analyses of chart-derived postoperative leakage and uncommon sling morphologies. Because no unmarked or alternative-imaging comparator was available, the study was not designed to establish superiority, diagnostic validity, prognostic thresholds or clinical effectiveness.

## 2. Materials and Methods

### 2.1. Study Design and Participants

This was a single-center, retrospective, cross-sectional methodological imaging study. The study population comprised consecutive women who underwent TVT-O surgery for female SUI and received standardized postoperative pelvic floor ultrasound between January 2024 and February 2026.

Inclusion criteria were as follows: (1) TVT-O surgery performed for female SUI; (2) postoperative transrectal biplane ultrasound examination; (3) availability of postoperative pelvic floor ultrasound images acquired and stored according to the study protocol; and (4) image quality sufficient to identify the sling, internal urethral orifice and cotton-swab-marked external urethral orifice, and to complete the main static sling-position measurements.

Exclusion criteria were as follows: (1) history of urinary tract tumor or pelvic malignancy; (2) history of major urethral reconstruction or complex urethral surgery; (3) absence of original postoperative pelvic floor ultrasound images or key clinical data; (4) inadequate image quality preventing identification of the sling or key anatomical reference points; and (5) any other condition judged by the investigators to be unsuitable for inclusion in this imaging analysis.

### 2.2. Surgical Information

Procedures were performed by two senior surgeons (associate chief physician or above) with extensive experience in female pelvic floor surgery. The surgical procedure was TVT-O. A longitudinal incision was made in the vaginal wall approximately 2 cm from the external urethral orifice, the vesicovaginal space was dissected toward the bilateral obturator routes, and the sling was placed beneath the mid-urethra through the obturator passage without tension. Excess sling material was trimmed, and the vaginal fascia and mucosa were closed. The sling used was the Gynecare TVT-O™ tension-free vaginal tape system (Ethicon, Inc., Somerville, NJ, USA), inserted using the inside-out approach. Concomitant procedures are shown in Table 1.

### 2.3. Ultrasound Examination

Pelvic floor ultrasound was performed using a Mindray Resona R9T system (Shenzhen Mindray Bio-Medical Electronics Co., Ltd., Shenzhen, China) with a 3D volume transducer (1.3–8.2 MHz), a biplane endocavitary transducer (4.8–11.0 MHz) and a convex transducer (3.5–9.5 MHz). Seven attending physicians or above, each with specialist training and at least 1000 pelvic floor examinations, acquired images under one institutional standard operating procedure. Training included patient positioning, external swab placement, imaging-plane selection, Valsalva coaching and image storage. One pelvic floor ultrasound specialist performed central quality control; images failing prespecified visualization requirements were excluded. Two designated physicians subsequently performed blinded offline measurements independently of the acquisition operators. Thus, the reliability analysis evaluates offline measurement, not between-operator acquisition variability.

Participants were examined in dorsal lithotomy with the bladder moderately filled (approximately 50–200 mL, without a strong urge to void). Under the same operating procedure, a paraffin-oil-lubricated cotton-swab tip was placed lightly against the external urethral orifice until a stable hyperechoic distal landmark was visible. The tip remained external: it was not advanced into the urethral lumen. Operators were instructed to avoid pressure that visibly displaced the distal urethra or anterior vaginal wall. Post-void residual urine volume was not measured uniformly in this retrospective cohort and was therefore not analyzed as an outcome.

Transrectal biplane ultrasound was then used to obtain sagittal and transverse images of the urethra, sling and anterior vaginal wall at rest and, when clinically appropriate, during maximum Valsalva. This route was selected for the present feasibility protocol because the high-frequency biplane probe provided simultaneous orthogonal views and allowed the external swab marker, urethra, sling and anterior vaginal wall to be depicted in the same examination. Transperineal/translabial and transvaginal ultrasound remained the first-line routes in routine practice; the present protocol was an investigational complementary examination. When several maneuvers were available, the image with maximal pelvic floor descent and adequate sling visualization was selected. Under the institutional postoperative safety workflow, Valsalva imaging was generally deferred during the first 3 months after surgery to avoid provoking discomfort or strain during early tissue healing. This planned omission was a principal source of systematic missingness in V-D-O and V-LA-A.

### 2.4. Ultrasound Measurements

**Figure 1:**
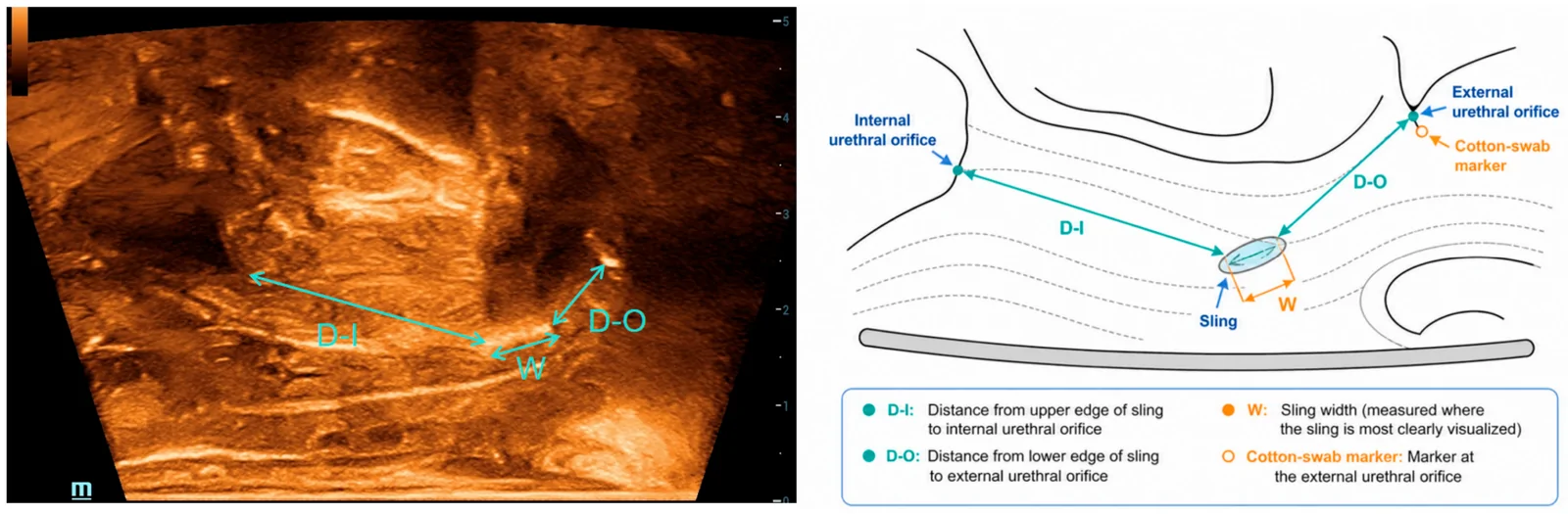
Representative transrectal biplane high-frequency ultrasound image and schematic illustration of the main measurement parameters. The sagittal two-dimensional B-mode image at rest (**left**) and the corresponding schematic (**right**) show the sling, internal urethral orifice and cotton-swab-marked external urethral orifice. W is the maximum visible sling width; D-I is the linear distance from the upper edge of the sling to the internal urethral orifice at rest; D-O is the linear distance from the lower edge of the sling to the marked external urethral orifice at rest; and V-D-O is the corresponding distance during maximum Valsalva maneuver.

The main measurements were as follows (Figure 1): (1) sling width (W), defined as the maximum width of the visible hyperechoic sling measured on the plane in which the sling was most clearly displayed; (2) D-I, the linear distance from the upper edge of the sling to the internal urethral orifice at rest; (3) D-O, the linear distance from the lower edge of the sling to the cotton-swab-marked external urethral orifice at rest; and (4) V-D-O, the linear distance from the lower edge of the sling to the cotton-swab-marked external urethral orifice during maximum Valsalva maneuver. All distances were recorded in millimeters. Established tape–urethra distance and proportional urethral position were not included because they were not prespecified in the image-review form and the standardized plane and calibration needed for reliable retrospective reconstruction were not preserved in every examination. These variables were therefore not reconstructed post hoc.

When available, levator hiatus area at rest and during Valsalva (LA-A and V-LA-A) was recorded only as a background measure of pelvic floor deformation and Valsalva performance. Because levator hiatus area is principally relevant to pelvic organ prolapse rather than a sling-specific urinary incontinence marker, these measurements were not interpreted as markers of continence.

In addition to quantitative measurements, uncommon sling appearances and relationships with adjacent structures were recorded qualitatively, including bilateral position asymmetry, folding or wrinkling, left–right length asymmetry and suspected anterior vaginal wall involvement. These categories were not prespecified with validated diagnostic thresholds and were not subjected to observer-agreement testing. They were therefore analyzed as post hoc exploratory observations only.

### 2.5. Clinical Outcome Variable

Postoperative leakage status was abstracted from routine records and defined as documentation of patient-reported involuntary urine leakage on coughing, straining or positional change, or urinary incontinence during daily activities at follow-up. This chart-derived binary variable was not a validated endpoint and may not distinguish persistent SUI from urgency urinary incontinence or other leakage phenotypes with sufficient accuracy [13].

Validated patient-reported instruments (e.g., ICIQ-UI SF), cough-stress testing, pad testing, post-void residual volume and urodynamic assessment were not applied uniformly. The leakage analysis was therefore prespecified in the revised interpretation as exploratory and hypothesis-generating; null associations were not treated as evidence of no clinically relevant relationship.

### 2.6. Interobserver Agreement

A computer-generated random sequence was created using IBM SPSS Statistics (version 26.0), and 40 cases were selected from the 112-patient cohort by simple random sampling without replacement, ensuring an equal probability of selection for each case. Two uniformly trained pelvic floor ultrasound physicians (physician A and physician B) independently measured the same set of images under mutual blinding (each was unaware of the other’s measurements) and were also blinded to patients’ clinical outcomes and the identity of the image-acquisition operator.

### 2.7. Statistical Analysis

Continuous variables were assessed with the Shapiro–Wilk test and visual distribution checks. Normally distributed data are reported as mean ± SD and compared using Welch’s *t*-test; non-normally distributed data are reported as median (IQR) and compared using the Mann–Whitney U-test. Paired non-normal measurements were compared using the Wilcoxon signed-rank test, with the Hodges–Lehmann median difference and 95% CI. Categorical variables are reported as *n* (%) and compared using Fisher’s exact test when expected counts were small. The actual denominator is reported for each variable.

Interobserver reliability was assessed using ICC(2,1): a two-way random-effects, absolute-agreement, single-measurement model [14]. ICCs were interpreted according to Koo and Li. Agreement and measurement error were additionally quantified using Bland–Altman mean bias (reader A minus reader B) with 95% LoA (bias ± 1.96 × SD of paired differences), SEM = SD of paired differences/√2, and MDC95 = 1.96 × SD of paired differences. Intraobserver reliability could not be estimated because same-reader repeated measurements were not collected; no within-reader repeatability estimate was imputed or inferred from the interobserver data.

Each analysis used all observations available for the variables involved. To assess possible selection bias, women with and without V-D-O were compared on age, postoperative interval, static ultrasound measures and leakage status. Holm adjustment controlled family-wise error across these six missingness comparisons. For the exploratory leakage analysis, six separate logistic regression models estimated the association of each ultrasound parameter with leakage after adjustment for age; Holm correction was applied to the six ultrasound *p* values. Adjusted odds ratios (ORs) with 95% CIs, raw *p* values and Holm-adjusted *p* values are reported. No imputation was performed because the principal Valsalva missingness was design-related and plausibly not missing at random. Analyses used IBM SPSS Statistics 26.0 and Python 3.12 (SciPy 1.16.3 and statsmodels 0.14.6). All tests were two-sided.

## 3. Results

### 3.1. Study Population and Data Availability

A total of 112 women were included. W, D-I and D-O were measurable in all 112 (100.0%); V-D-O was available in 95 (84.8%), LA-A in 63 (56.3%) and V-LA-A in 46 (41.1%). Postoperative leakage status was available in 101 women (90.2%): 62 had no documented leakage and 39 had leakage. Age was available in 111 and the postoperative interval in 91. Compared with women with V-D-O, those without V-D-O had a shorter postoperative interval (median, 84 vs. 106 days; raw *p* = 0.0027; Holm-adjusted *p* = 0.016). Age showed a raw difference (44 vs. 51 years; *p* = 0.0446) that did not persist after correction (adjusted *p* = 0.223). Static measures and leakage status did not differ after Holm correction. These findings support systematic rather than completely random V-D-O missingness and limit generalization of the dynamic analysis to early postoperative patients.

### 3.2. Main Ultrasound Measurements

Table 2 presents all ultrasound variables consistently as median (IQR), with variable-specific denominators. In the 101 women with leakage status, none of the six unadjusted comparisons reached statistical significance. These cross-sectional comparisons are exploratory because leakage was chart-derived and follow-up timing was heterogeneous.

### 3.3. Dynamic Changes During Valsalva Maneuver

**Figure 2:**
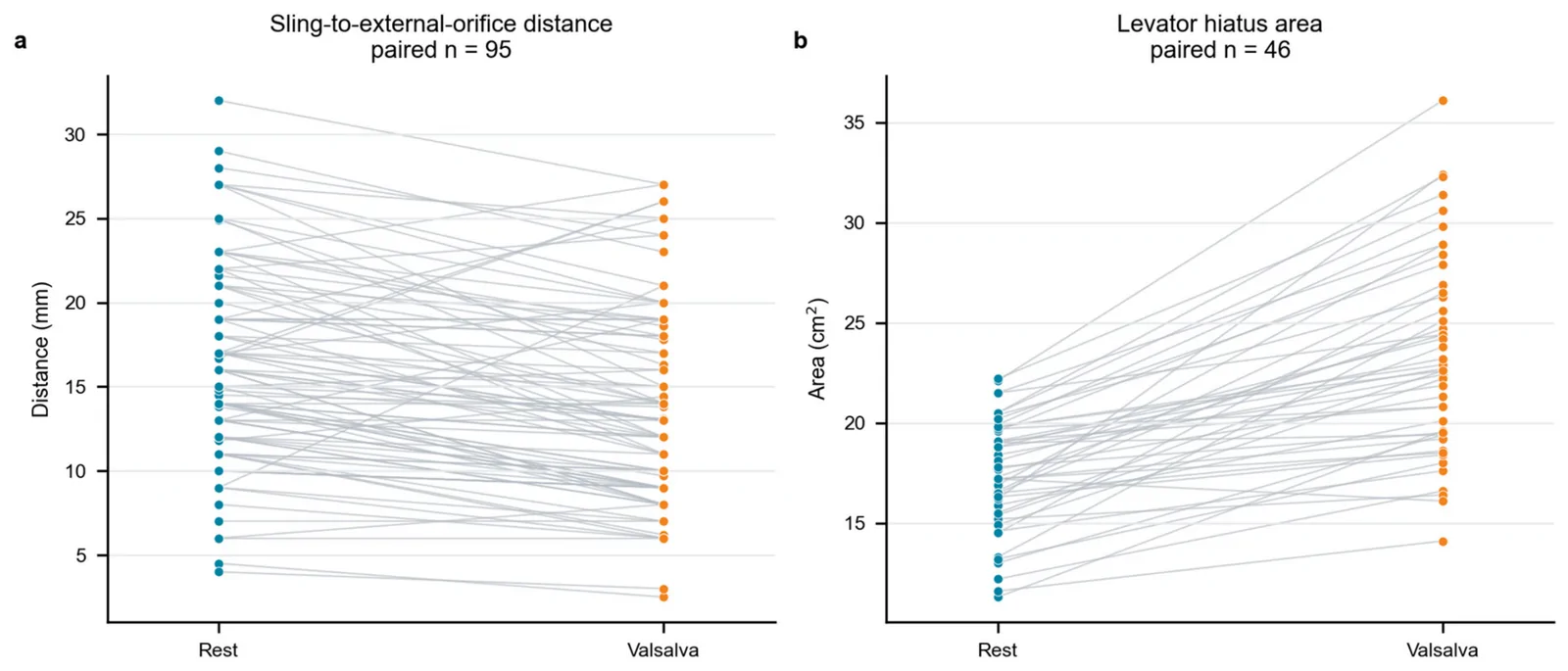
Paired changes from rest to maximum Valsalva. (**a**) D-O and V-D-O (*n* = 95; Wilcoxon signed-rank *p* < 0.001). (**b**) LA-A and V-LA-A (*n* = 46; Wilcoxon signed-rank *p* < 0.001). Thin lines connect measurements from the same woman; thick black lines show group medians. LA-A was included as a background measure of pelvic floor deformation, not as a sling-specific continence marker.

In 95 women with paired D-O and V-D-O, the distal sling-to-orifice distance was shorter during Valsalva than at rest (16.00 [12.00–20.00] vs. 13.00 [9.00–18.00] mm; Hodges–Lehmann median difference, −3.00 mm [95% CI, −4.90 to −1.00]; *p* < 0.001). In 46 women with paired LA-A and V-LA-A, levator hiatus area increased during Valsalva (18.10 [15.95–20.05] vs. 22.65 [19.42–26.45] cm^2^; *p* < 0.001) (Figure 2). The latter finding indicates pelvic floor deformation during straining and is not interpreted as a marker of urinary continence.

### 3.4. Exploratory Comparison According to Postoperative Leakage Symptoms

**Figure 3:**
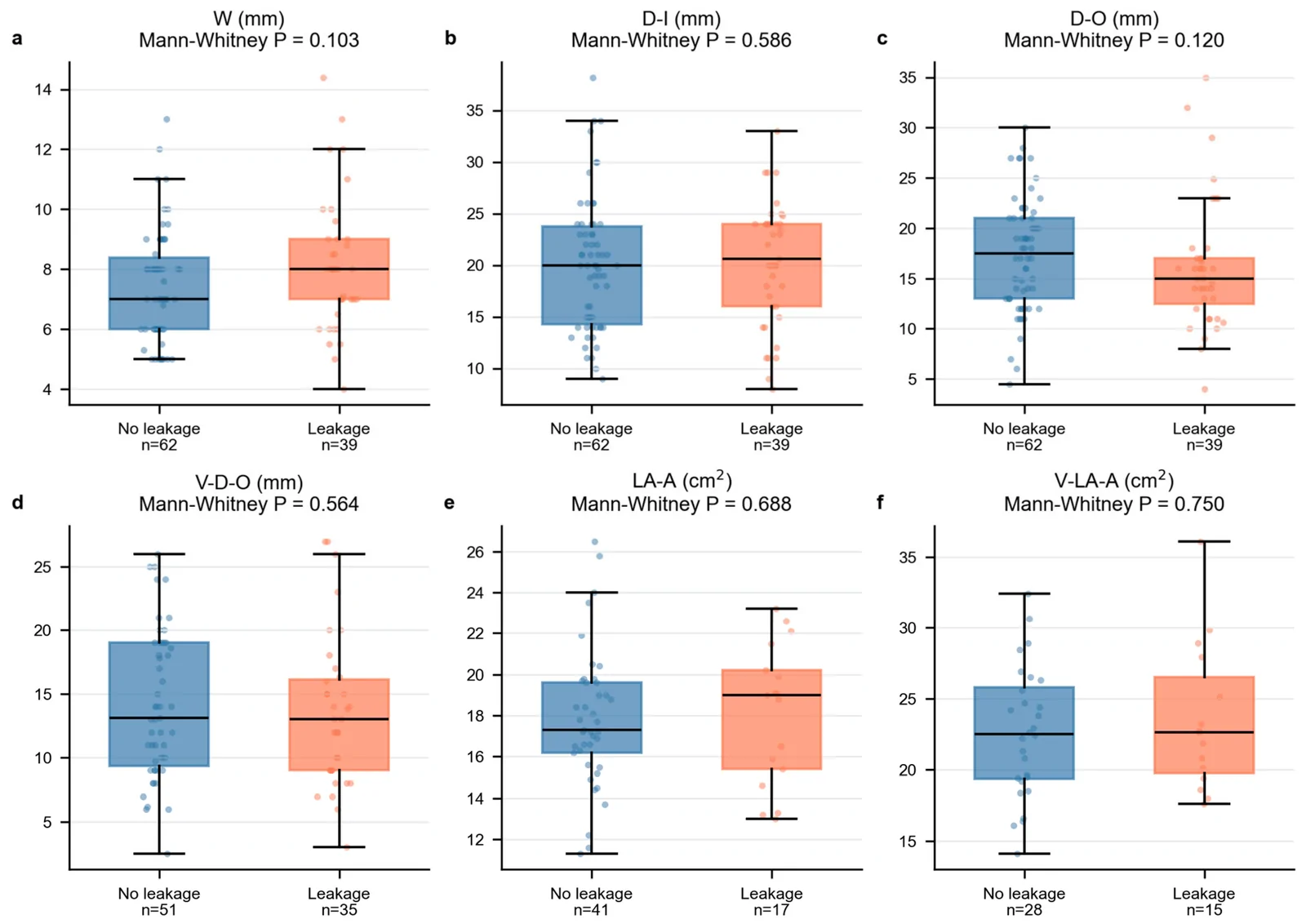
Exploratory ultrasound comparisons according to chart-derived postoperative leakage status. (**a**) Sling width (W); (**b**) distance from the upper edge of the sling to the internal urethral orifice at rest (D-I); (**c**) distance from the lower edge of the sling to the cotton-swab-marked external urethral orifice at rest (D-O); (**d**) corresponding distance during maximum Valsalva maneuver (V-D-O); (**e**) levator hiatus area at rest (LA-A); (**f**) levator hiatus area during Valsalva maneuver (V-LA-A). Boxes show the median and interquartile range (IQR); whiskers extend to 1.5 × IQR; points show individual observations. Panel-specific sample sizes and raw two-sided Mann–Whitney U p values are displayed. Multiple-testing correction was applied in the age-adjusted models reported in Table 3, not to these descriptive plots.

Leakage status was available in 101 women (62 without and 39 with leakage). Unadjusted comparisons of W, D-I, D-O, V-D-O, LA-A and V-LA-A were all non-significant (Table 2; Figure 3). In separate age-adjusted logistic models, the ORs per unit increase were 1.18 for W, 1.02 for D-I, 0.97 for D-O, 0.98 for V-D-O, 1.01 for LA-A and 1.04 for V-LA-A; no ultrasound term remained significant after Holm correction (all adjusted *p* ≥ 0.773; Table 3). These null exploratory results are imprecise and should not be interpreted as evidence that sling position is unrelated to outcome.

### 3.5. Interobserver Reliability and Measurement Error

**Figure 4:**
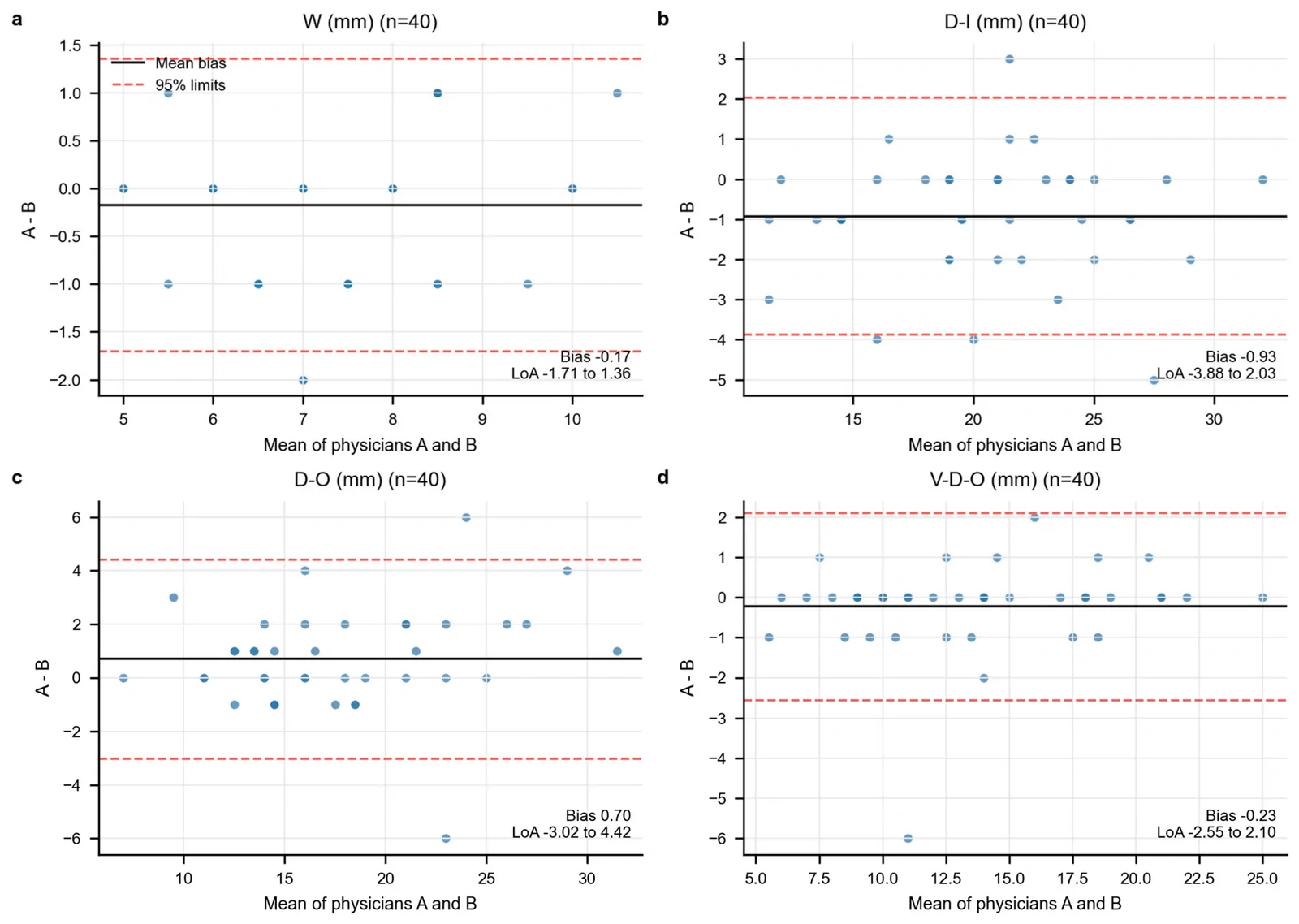
Bland–Altman assessment of interobserver agreement (n = 40 paired measurements per parameter). The solid line is the mean bias (reader A minus reader B), and dashed lines are the 95% limits of agreement. ICC(2,1), bias and limits are displayed in each panel. W, sling width; D-I, sling upper edge to internal urethral orifice; D-O/V-D-O, sling lower edge to externally marked urethral orifice at rest/during Valsalva.Bland–Altman assessment of interobserver agreement (n = 40 paired measurements per parameter). (**a**) Sling width (W); (**b**) distance from the upper edge of the sling to the internal urethral orifice at rest (D-I); (**c**) distance from the lower edge of the sling to the cotton-swab-marked external urethral orifice at rest (D-O); (**d**) corresponding distance during maximum Valsalva maneuver (V-D-O). In each panel, the solid line represents the mean bias (reader A minus reader B), and the dashed lines represent the 95% limits of agreement. ICC(2,1), mean bias, and 95% limits of agreement are displayed in each panel.

Forty randomly selected examinations were measured independently by two blinded readers. ICCs were good for W (0.845) and excellent for D-I (0.942), D-O (0.936) and V-D-O (0.970). Bland–Altman biases were −0.18, −0.93, 0.70 and −0.23 mm, respectively. The corresponding 95% LoA were −1.70 to 1.36, −3.88 to 2.03, −3.02 to 4.42 and −2.55 to 2.10 mm; MDC95 values were 1.53, 2.96, 3.72 and 2.33 mm (Table 4; Figure 4). These error bounds describe agreement between offline readers but do not establish clinical validity or acquisition reproducibility.

### 3.6. Exploratory Sling Morphology

Post hoc review identified bilateral sling-position asymmetry in 2/112 women (1.8%), folding or wrinkling in 3/112 (2.7%), left–right sling-length asymmetry in 1/112 (0.9%) and suspected anterior vaginal wall involvement in 1/112 (0.9%). Two of three women with folding/wrinkling had chart-documented leakage. Because definitions were not prespecified, counts were very small and agreement was not tested; these observations are illustrative only and cannot support diagnostic or causal inference.

## 4. Discussion

### 4.1. Principal Findings

This study supports technical feasibility and offline interobserver reliability of a complementary transrectal biplane protocol with external cotton-swab landmarking after TVT-O. Static measures were obtainable in all 112 women, and four primary distances showed good-to-excellent ICCs with quantified Bland–Altman error bounds. Valsalva measurements demonstrated dynamic change but were systematically less available early after surgery. Age-adjusted exploratory models did not identify associations with chart-derived leakage. The results do not establish superiority over transperineal/translabial or transvaginal ultrasound, measurement validity, prognostic utility or clinical benefit.

### 4.2. Methodological Implications

A stable anatomical reference is important for repeatable sling measurement. Previous studies have used the bladder neck, urethral length, pubic symphysis or periurethral structures, but methods vary [7,8,9,10,11,15]. External swab contact made the external urethral orifice visible in the stored examinations and supported high measurement availability. However, because no unmarked scan was acquired in the same patients, the present data cannot show that marking improves visibility, precision or standardization relative to conventional imaging. Contact pressure and operator placement may also alter local anatomy and require prospective testing.

Transperineal/translabial and transvaginal ultrasound remain the literature-supported first-line modalities for postoperative sling imaging [3,4,5,6,12]. Transrectal biplane imaging is not included as an established first-line route in these sources and should not be interpreted as a substitute. Its potential role in this study is therefore limited to an investigational complementary view when additional sagittal/transverse detail is sought, and the balance of added information, discomfort, infection-control burden and feasibility must be evaluated directly before routine clinical use.

### 4.3. Relationship with Previous Studies

Prior ultrasound studies have linked sling position, tape–urethra distance and functional configuration with continence or complications [7,8,9,10,11,15,16,17,18,19,20,21,22,23,24,25,26,27,28,29,30]. Kociszewski and colleagues in particular described tape functionality, tape–urethra distance and proportional urethral position [20,21]. Those measures were not prospectively specified in our protocol and cannot be reconstructed reliably from all stored images because the required standardized plane and calibration were not consistently preserved. We therefore did not derive them retrospectively, have added this omission as a limitation and identify them as mandatory comparator measures for future prospective work.

The present study was designed to assess feasibility and reliability, not to establish a threshold for cure or recurrence. Chart-derived leakage is susceptible to outcome misclassification, timing heterogeneity and confounding. Even after age adjustment and Holm correction, the absence of statistical associations remains inconclusive because sample sizes were modest and residual confounding was not addressed. The findings neither confirm nor refute the clinical value of established sling ultrasound measurements.

### 4.4. Interpretation of Dynamic Changes During Valsalva Maneuver

The shorter V-D-O during Valsalva indicates measurable relative displacement between the sling edge and an external distal urethral landmark; it does not identify movement of either structure in isolation. Expansion of the levator hiatus confirmed pelvic floor deformation during straining but was recorded as contextual information rather than as a urinary-incontinence marker. Because Valsalva was commonly deferred during the first 3 postoperative months, this dynamic result applies primarily to later follow-up and may be biased if early postoperative mechanics differ.

The externally positioned marker remained visible during many dynamic examinations, but this observation alone does not prove improved standardization. A rigorous validation should compare marked and unmarked acquisitions within the same participants, randomize acquisition order, quantify discomfort and adverse effects, and evaluate both acquisition- and measurement-level reliability.

### 4.5. Clinical Significance of Abnormal Sling Morphology

**Figure 5:**
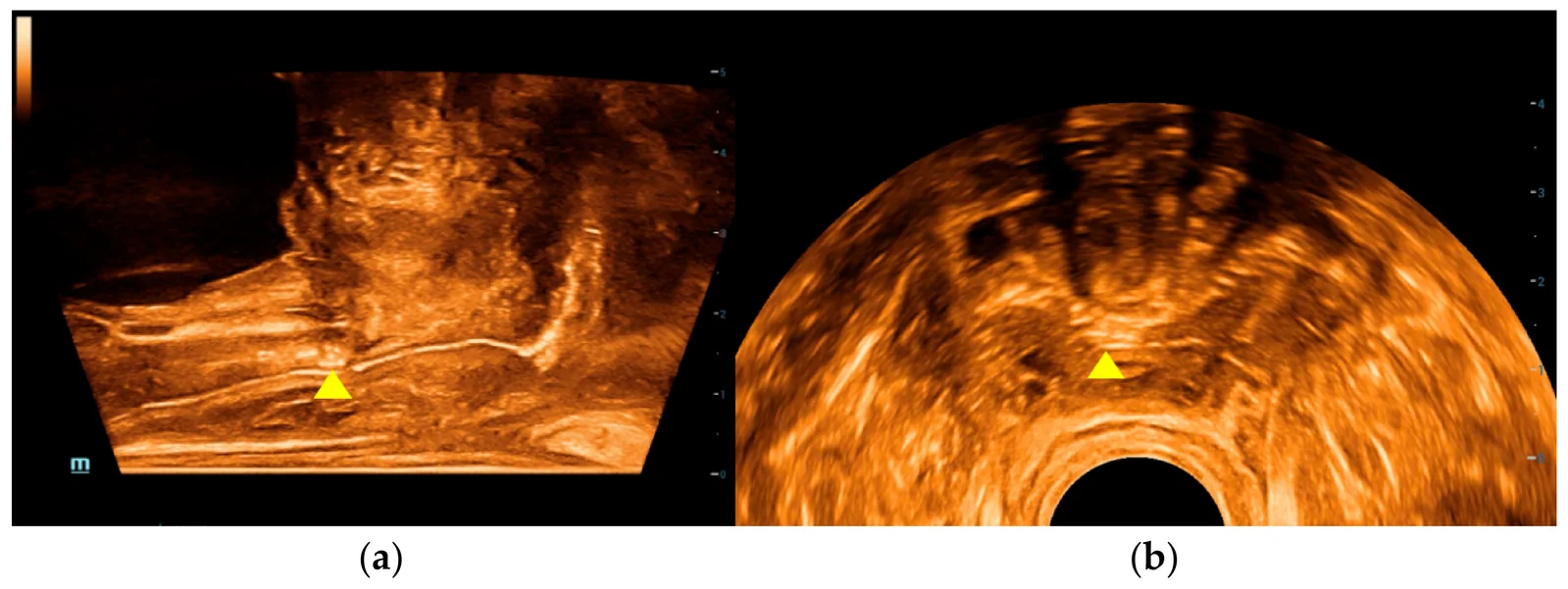
Suspected sling involvement of the anterior vaginal wall on transrectal biplane high-frequency ultrasound. Annotated sagittal (**a**) and transverse (**b**) two-dimensional B-mode images show the suspected sling–anterior vaginal wall interface (the site indicated by the yellow triangles). The appearance is illustrative and does not establish tissue involvement diagnostically.

**Figure 6:**
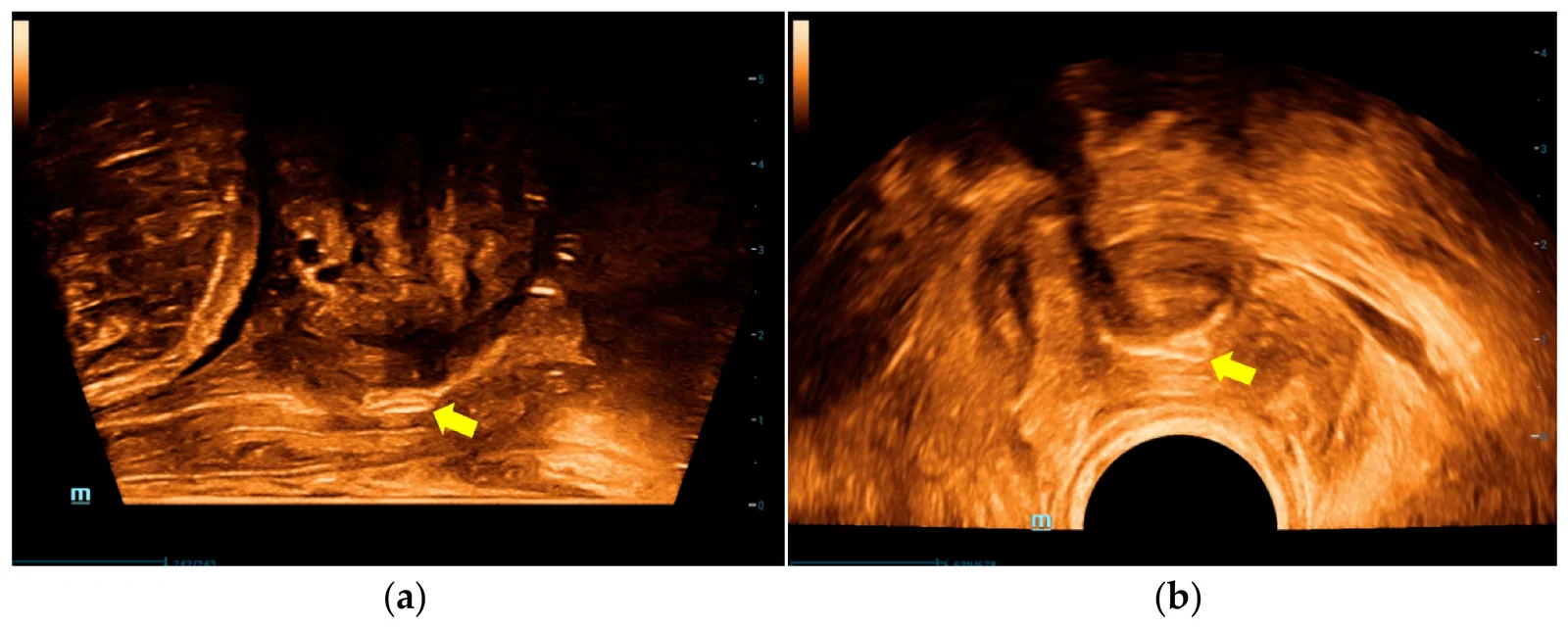
Annotated transrectal biplane ultrasound example of sling folding/wrinkling (the site indicated by the yellow arrows). (**a**) Sagittal and (**b**) transverse views. The image is presented for anatomical orientation and descriptive illustration only.

Sling folding, asymmetry and possible tissue involvement may be clinically relevant, but our post hoc observations lacked prespecified criteria, adequate event counts and observer testing. Figure 5 and Figure 6 are therefore illustrative. No relationship with leakage, pain, erosion or voiding dysfunction should be inferred from these cases.

### 4.6. Clinical Implications

The protocol may provide a structured research workflow for offline measurement and anatomical illustration. It is not a validated postoperative assessment tool and should not displace first-line transperineal/translabial or transvaginal ultrasound. Clinical evaluation should integrate symptoms, examination, cough-stress testing, pad testing, post-void residual volume when indicated, validated questionnaires, urodynamics and operative information.

The quantified LoA and MDC95 help define the smallest between-reader differences distinguishable from measurement error in this dataset, but no clinical thresholds have been validated. Individual values should not currently be used to diagnose failure, select reoperation or predict recurrence.

### 4.7. Strengths

Strengths include consecutive case inclusion, a clearly described external marking procedure, complete availability of three static measurements, blinded independent measurement of a random subset, and extension of reliability reporting beyond ICCs to Bland–Altman limits and error metrics. The revised analysis also reports variable-specific denominators, evaluates a key missingness pattern and uses age-adjusted sensitivity models with multiplicity correction.

### 4.8. Limitations

This study has important limitations. It was retrospective and single-center. Leakage was chart-derived rather than measured with validated instruments or objective testing, and follow-up timing varied. Valsalva and levator-hiatus data were incomplete; missing V-D-O was associated with shorter postoperative interval, so complete-case dynamic estimates may not generalize to early follow-up. There was no unmarked, transperineal/translabial or transvaginal comparator, precluding claims of superiority or criterion validity. Same-reader repeated measurements were unavailable, so intraobserver reliability could not be estimated. Seven physicians acquired images; offline reader agreement does not quantify acquisition variability. Tape–urethra distance and proportional urethral position were not prospectively specified and could not be reconstructed reliably from every stored examination; post-void residual volume was not recorded consistently. Qualitative morphology was post hoc without prespecified definitions or reliability testing. Finally, swab contact and a transrectal probe may cause discomfort, alter local anatomy or affect Valsalva performance; these effects were not quantified.

### 4.9. Future Research

Prospective multicenter validation should compare external marking with unmarked transperineal/translabial and transvaginal acquisitions within the same women, use randomized acquisition order, and evaluate discomfort, safety, image adequacy and operator-level variability. Uniform follow-up should include ICIQ-UI SF, cough-stress testing, pad testing, post-void residual volume and urodynamic assessment when indicated [1].

Future protocols should include repeated same-reader measurements, prespecified acceptable LoA/MDC thresholds, independent validation, and established tape–urethra distance and proportional-position measures described by Kociszewski and others [20,21,22,23,24,25,26,27,28,29,30]. Morphological criteria should be defined before image review and tested for inter- and intraobserver reliability. These steps are required before the method can be evaluated for diagnostic or prognostic use.

## 5. Conclusions

Transrectal biplane ultrasound with external cotton-swab marking permitted highly available static measurements and good-to-excellent offline interobserver reliability in this retrospective cohort. Bland–Altman analysis quantified between-reader error, while missingness analysis showed that Valsalva measurements were less available early after surgery. The protocol should be regarded as a feasibility and reliability method only. Its incremental value over first-line transperineal/translabial or transvaginal ultrasound, clinical validity and prognostic utility remain unproven and require prospective comparative validation.

## Figures and Tables

**Table 1:** Baseline characteristics according to postoperative leakage status.

Characteristic	Overall	No Leakage	Leakage	*p* Value
Available leakage status, n	101	62	39	—
Age, years	49.88 ± 8.66 (N = 101)	48.44 ± 8.88 (*n* = 62)	52.18 ± 7.85 (*n* = 39)	0.029
Postoperative interval, days	102 (92, 165) (*n* = 81)	99 (92, 123) (*n* = 49)	116.5 (94.25, 433.25) (*n* = 32)	0.053
Comprehensive pelvic floor reconstruction	64/101 (63.4)	40/62 (64.5)	24/39 (61.5)	0.833
Anterior/posterior vaginal wall repair	15/101 (14.9)	7/62 (11.3)	8/39 (20.5)	0.254
Apical suspension	11/101 (10.9)	3/62 (4.8)	8/39 (20.5)	0.021
Hysterectomy	8/101 (7.9)	3/62 (4.8)	5/39 (12.8)	0.255
Perineoplasty/vulvoplasty	9/101 (8.9)	6/62 (9.7)	3/39 (7.7)	1.000
Other postoperative symptoms	24/97 (24.7)	18/59 (30.5)	6/38 (15.8)	0.148
Abnormal sling morphology	8/101 (7.9)	5/62 (8.1)	3/39 (7.7)	1.000

Data are presented as mean ± SD, median (IQR), or *n*/N (%), as appropriate. *p* values are from Welch’s *t*-test for age, Mann–Whitney U-test for postoperative interval and Fisher’s exact test for categorical variables. Denominators vary because of missing chart data. “Comprehensive pelvic floor reconstruction” denotes multi-compartment reconstructive surgery recorded in the operative note; specific concomitant procedures are listed separately and categories may overlap. Other postoperative symptoms included storage lower urinary tract symptoms, voiding/postmicturition symptoms, pain or discomfort, urinary tract infection, dyspareunia or bleeding. Leakage was a chart-derived exploratory endpoint; post-void residual volume and validated continence tests were not uniformly available.

**Table 2:** Ultrasound measurements according to postoperative leakage status.

Ultrasound Measurement	Overall	No Leakage	Leakage	Raw *p*
W, mm	7.35 (6.00, 9.00) (n = 112)	7.00 (6.00, 8.38) (n = 62)	8.00 (7.00, 9.00) (n = 39)	0.103
D-I, mm	20.30 (15.00, 24.00) (n = 112)	20.00 (14.25, 23.75) (n = 62)	20.60 (16.00, 24.00) (n = 39)	0.586
D-O, mm	16.00 (12.00, 20.00) (n = 112)	17.50 (13.00, 21.00) (n = 62)	15.00 (12.50, 17.00) (n = 39)	0.120
V-D-O, mm	13.00 (9.00, 18.00) (n = 95)	13.10 (9.35, 19.00) (n = 51)	13.00 (9.00, 16.15) (n = 35)	0.564
LA-A, cm^2^	18.10 (15.95, 20.05) (n = 63)	17.30 (16.20, 19.60) (n = 41)	19.00 (15.40, 20.20) (n = 17)	0.688
V-LA-A, cm^2^	22.65 (19.42, 26.45) (n = 46)	22.50 (19.35, 25.78) (n = 28)	22.60 (19.75, 26.50) (n = 15)	0.750

Data are median (IQR). *p* values are from two-sided Mann–Whitney U-tests and are unadjusted descriptive comparisons. Denominators are reported for each group because Valsalva and levator-hiatus measurements were incomplete.

**Table 3:** Age-adjusted exploratory logistic regression models for chart-derived postoperative leakage.

Parameter	n	Adjusted OR (95% CI)	Raw *p*	Holm-Adjusted *p*
W, per mm	101	1.178 (0.953–1.456)	0.129	0.773
D-I, per mm	101	1.016 (0.951–1.086)	0.639	1.000
D-O, per mm	101	0.970 (0.903–1.041)	0.398	1.000
V-D-O, per mm	86	0.983 (0.911–1.061)	0.661	1.000
LA-A, per cm^2^	58	1.013 (0.853–1.204)	0.883	1.000
V-LA-A, per cm^2^	43	1.043 (0.913–1.192)	0.537	1.000

**Table 4:** Interobserver reliability, Bland–Altman agreement and measurement error (n = 40).

Parameter	ICC (95% CI)	Bias, mm	95% LoA, mm	SEM, mm	MDC95, mm
W	0.845 (0.726–0.915)	−0.18	−1.70 to 1.36	0.55	1.53
D-I	0.942 (0.896–0.970)	−0.93	−3.88 to 2.03	1.07	2.96
D-O	0.936 (0.877–0.972)	0.70	−3.02 to 4.42	1.34	3.72
V-D-O	0.970 (0.944–0.984)	−0.23	−2.55 to 2.10	0.84	2.33

## Data Availability

The participant-level dataset is not publicly available because it contains potentially identifiable clinical information and is subject to institutional ethics and data-protection requirements. De-identified data supporting the findings may be made available by the corresponding author on reasonable request, subject to institutional approval.

## Abbreviations

The following abbreviations are used in this manuscript:

SUI	Stress urinary incontinence
TVT-O	Tension-free vaginal tape-obturator
W	Sling width
D-I	Linear distance from the upper edge of the sling to the internal urethral orifice at rest
D-O	Linear distance from the lower edge of the sling to the cotton-swab-marked external urethral orifice at rest
V-D-O	Linear distance from the lower edge of the sling to the cotton-swab-marked external urethral orifice during maximum Valsalva maneuver
LA-A	Levator hiatus area at rest
V-LA-A	Levator hiatus area during Valsalva maneuver
